# *Arabidopsis* YL1/BPG2 Is Involved in Seedling Shoot Response to Salt Stress through ABI4

**DOI:** 10.1038/srep30163

**Published:** 2016-07-22

**Authors:** Peng-Cheng Li, Jin-Guang Huang, Shao-Wei Yu, Yuan-Yuan Li, Peng Sun, Chang-Ai Wu, Cheng-Chao Zheng

**Affiliations:** 1 State Key Laboratory of Crop Biology, College of Life Sciences, Shandong Agricultural University, Tai’an, Shandong 271018, PR China; 2 Bio-Tech Research Center, Shandong Academy of Agricultural Sciences, Shandong Provincial Key Laboratory of Crop Genetic Improvement, Ecology and Physiology, Jinan, Shandong, PR China

## Abstract

The chloroplast-localized proteins play roles in plant salt stress response, but their mechanisms remain largely unknown. In this study, we screened a yellow leaf mutant, *yl1-1*, whose shoots exhibited hypersensitivity to salt stress. We mapped *YL1* to AT3G57180, which encodes a YqeH-type GTPase. YL1, as a chloroplast stroma-localized protein, could be markedly reduced by high salinity. Upon exposure to high salinity, seedling shoots of *yl1-1* and *yl1-2* accumulated significantly higher levels of Na^+^ than wild type. Expression analysis of factors involved in plant salt stress response showed that the expression of *ABI4* was increased and *HKT1* was evidently suppressed in mutant shoots compared with the wild type under normal growth conditions. Moreover, salinity effects on *ABI4* and *HKT1* were clearly weakened in the mutant shoots, suggesting that the loss of YL1 function impairs *ABI4* and *HKT1* expression. Notably, the shoots of *yl1-2 abi4* double mutant exhibited stronger resistance to salt stress and accumulated less Na^+^ levels after salt treatment compared with the *yl1-2* single mutant, suggesting the salt-sensitive phenotype of *yl1-2* seedlings could be rescued via loss of ABI4 function. These results reveal that YL1 is involved in the salt stress response of seedling shoots through ABI4.

High salinity is a serious factor that influences plant productivity. It affects various aspects of plant physiology and metabolism by inducing osmotic stress and ion toxicity^1^. The early-occurring osmotic stress triggers physiological changes, such as membrane interruption in roots and reduction of water absorption capacity in plants. Ion over-accumulation, which is the second phase of salt stress, can induce severe Na^+^/K^+^ imbalance and toxic effects^2^,^3^,^4^.

Plants have evolved different molecular mechanisms to adapt to hyperionic stress^4^,^5^. The calcium-responsive salt overly sensitive (SOS) regulatory pathway, which is mainly for ion homeostasis, has been established in *Arabidopsis*^6^. SOS3, a myristoylated calcium-binding protein, recognizes salt-elicited cytosolic calcium signals and then interacts with and activates SOS2 for signal transmission^7^,^8^. SOS1, a plasma membrane Na^+^/H^+^ anti-porter and regulated by SOS3 and SOS2, aids in sodium transport from root cells back into the soil or from epidermal cells into the xylem^9^. Overexpression of SOS1 confers plant salt stress resistance^10^. High-affinity K^+^ transporter (HKT1), an important regulator that is independent of the SOS pathway and exhibits significant functions in root and shoot ion homeostasis, has been extensively studied^11^,^12^. HKT1, as a K^+^/Na^+^ symporter, is highly expressed in the root stele and leaf vasculature and retrieves sodium from the root-to-shoot xylem sap in *Arabidopsis*^4^,^13^. *AtHKT1* knock-out mutant leaves exhibit high sensitivity to salt stress because of excessive sodium accumulation^14^, and *AtHKT1* overexpression in roots enhances the salt tolerance of the entire plant^15^. In addition, the tonoplast-localized Na^+^ (K^+^)/H^+^ exchanger NHX1 confers Na^+^ or K^+^ storage into vacuoles^16^,^17^. AtNHX1 overexpression could reduce Na^+^ stress through enhancing intracellular K^+^/Na^+^ ratios in tomato^18^.

The phytohormone abscisic acid (ABA) exerts a significant function for coping with salt stress^3^. The ABA-deficient mutants *aba1*, *aba2*, and *aba3* show a readily wilting phenotype under salt or drought stress. ABSCISIC ACID INSENSITIVE (ABI) 4 was first isolated from a screen for ABA-insensitive mutants during seed germination^19^. ABI4, as a member of the plant-specific AP2/EREBP family, is involved in many signal transduction pathways, such as sugar signaling and mitochondrial/chloroplast retrograde signaling^20^,^21^,^22^. The *abi4* mutant exhibits salt stress resistance because less sodium is accumulated in plant shoots. ABI4-overexpressing (dexamethasone-induced) plants show increased salt sensitivity because ABI4 downregulates *HKT1* expression by directly binding to the promoter ABE-element GC(C/G)GCTT(T)^23^.

It is generally accepted that, high salinity can cause photosynthesis inhibition in plants, and leaf growth is very sensitive to salt stress. This phenomenon may be attributed to the disruption of chloroplast development^24^,^25^. CO_2_ fixation is sensitive to environmental stresses. Therefore, salt stress can inhibit the repair of PS II via the ROS-induced suppression of PS II protein synthesis, which in turn triggers an imbalance between the photo-damage and repair rates of PS II^26^,^27^. Moreover, recent studies have suggested that the chloroplast proteins also play roles in plant salt stress response^28^,^29^,^30^. However, the mechanisms are largely unclear. In this study, we screened the *yellow leaf 1-1* (*yl1-1*) mutant. The shoots of *yl1-1* showed evident salt stress-sensitive phenotypes. We demonstrated that YL1, as a chloroplast protein, is involved in the high salinity response of seedling shoots through ABI4.

## Results

### Phenotypes of *yl1-1* Mutant

**Figure 1:**
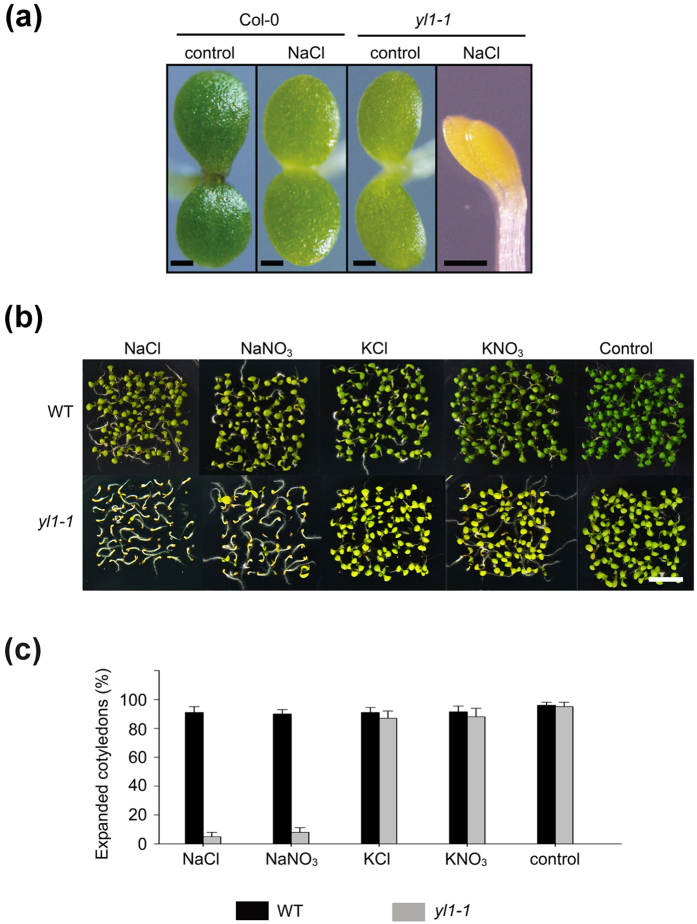
Salt stress sensitive phenotypes of *yl1-1* seedling shoots. (**a**) Phenotypes of seedlings under control or salt stress conditions. Each lane represents the corresponding seedling grown for 5 days after germination under the indicated condition (NaCl, 150 mM). Three independent experiments were performed with similar results and one representative is showed. Scale bars = 0.5 mm. (**b**) Phenotypes of wild type or *yl1-1* plants grown in different salinity stresses (NaCl, NaNO_3_, KCl, KNO_3_, and control; 150 mM) for 5 days after germination. Three independent experiments were performed with similar results and one representative is showed. Scale bar = 5 mm. (**c**) The percentages of the fully expanded seedling cotyledons showed in (**b)**. Values are means ± SE of three independent replicates.

*Arabidopsis* seedling shoots usually exhibit pale coloration and stunted phenotypes under salt stress conditions (Fig. 1a). We are interested in mutants that the seedling shoots exhibit extremely sensitive phenotypes under salt stress. The mutant *yl1-1* was isolated from approximately 30,000 ethane methylsulfonate (EMS)-mutagenized Col-0 M2 seedlings, which conferred a pale-green shoot phenotype under normal growth conditions (Figs 1a and S1). However, under salt stress conditions, shoot of *yl1-1* showed evidently stunted phenotype compared with wild type (Figs 1a and S1), while little differences in root development could be observed (Fig. S1). Three additional salts (NaNO_3_, KCl, or KNO_3_) were used in seedling growth experiments to understand the phenotypes of *yl1-1* hypersensitivity to salt stress better. The results showed that the percentages of the fully expanded cotyledons of *yl1-1* seedlings were significantly lower in growth conditions with NaCl or NaNO_3_ than with KCl or KNO_3_ (Fig. 1b,c). By contrast, wild type seedlings did not exhibit clear differences under these different salt treatments (Fig. 1b,c). These observations suggest that Na^+^ toxicity leads to stunted *yl1-1* shoot phenotypes.

### Positional Cloning of *yl1-1*

**Figure 2:**
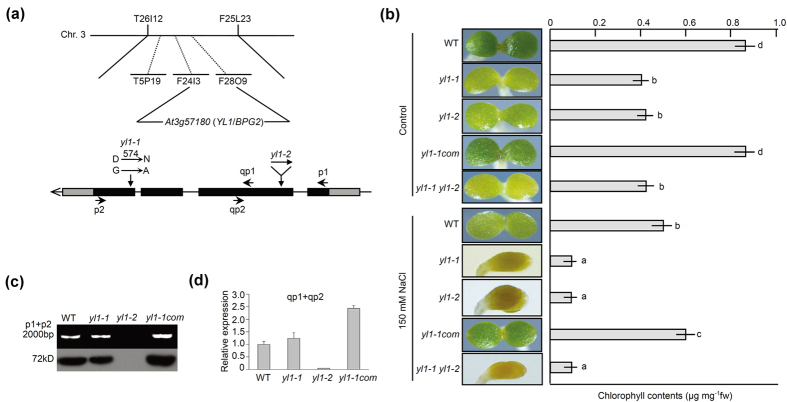
Positional cloning of *yl1-1*. (**a**) Map position of *yl1-1* on chromosome 3. Some of the used BAC markers are indicated. Gene structure of *YL1*(*At3g57180*, *BPG2*) is shown. The *yl1-1* mutated site of G to A (amino acid of D to N) and the inserted position of a *YL1* T-DNA insertion mutant, *yl1-2*, are all indicated. Short arrows represent primers used in (**c**,**d)**. (**b**) Phenotypes of wild type, *yl1-1*, *yl1-2*, *yl1-1com*, and *yl1-1 yl1-2* (cross F1 line) seedling grown under control or 150 mM NaCl condition for 5 days after germination. Scale bars = 0.5 mm. Endogenous chlorophyll (**a**,**b**) contents of the corresponding plants are shown. Data are mean values of three replicates ± SE. Statistical significant differences are indicated by different lowercase letters (P < 0.01). (**c**) Full-length *YL1* gene amplification from cDNA and immunoblot analysis for YL1 in wild type, *yl1-1*, *yl1-2*, and *yl1-1* complementary plants (*yl1-1com*). Primers p1 and p2 showed in **a** were used. (**d**) qRT-PCR analysis of *YL1* expression levels in wild type, *yl1-1*, *yl1-2*, and *yl1-1* complementary plant *yl1-1com*. Primers qp1 and qp2 showed in **a** were used. The internal control gene was *Actin2*.

A map-based cloning approach was applied to identify the target gene(s) responsible for the *yl1-1* phenotypes (Fig. 2a). F1 plants were generated by crossing *yl1-1* with Landsberg (an *Arabidopsis* wild ecotype) and self-fertilization to generate the F2 population. We mapped *YL1* to *At3g57180* (*Brassinazole Insensitive Pale Green 2*, *BPG2*), positioned it between the BAC markers F24I3 and F28O9, and identified a G to A base substitution, which caused a D to N change in position 574 of the amino acid sequence (Fig. 2a).

Previous studies reported that BPG2, as a YqeH-type GTPase, participates in chloroplast rRNA maturation^31^,^32^. To determine whether the lack of YL1 is responsible for the phenotypes of *yl1-1*, we introduced the full-length open reading frame of YL1 to the *yl1-1* mutant under the control of the *35S* promoter. The complemented plant (*yl1-1com*) showed a normal leaf phenotype (Figs 2b and S2a). A mutant of *At3g57180*, *yl1-2* (*bpg2-2*, salk_068713), was obtained from ABRC stock (Figs 2b and S2a). Western blot, gene full-length amplification PCR from cDNA, and real-time quantitative PCR (qRT-PCR) analyses showed that *YL1* expression was extremely low in *yl1-2*, which suggested a knock-out mutant (Fig. 2c,d). The *yl1-1*, *yl1-2*, and cross F1 line (*yl1-1 yl1-2*) exhibited evident pale-green leaf phenotypes under normal growth conditions (Figs 2b and S2a). Under salt stress treatment, the *yl1-1*, *yl1-2*, and cross F1 line showed extremely stunted cotyledon phenotypes, whereas the wild type and *yl1-1com* plants exhibited normal phenotypes except for the decrease of chlorophyll contents at day 5 after germination (Fig. 2b). In addition, the F_v_/F_m_ and endogenous contents of chlorophyll in the mature plants of *yl1-1*, *yl1-2*, and cross F1 line were significantly lower than those in the wild type and *yl1-1com* (Fig. S2b,c). These results indicate that the *yl1-1* phenotypes are caused by YL1 function loss.

### Subcellular Localization and Expression Pattern of YL1

**Figure 3:**
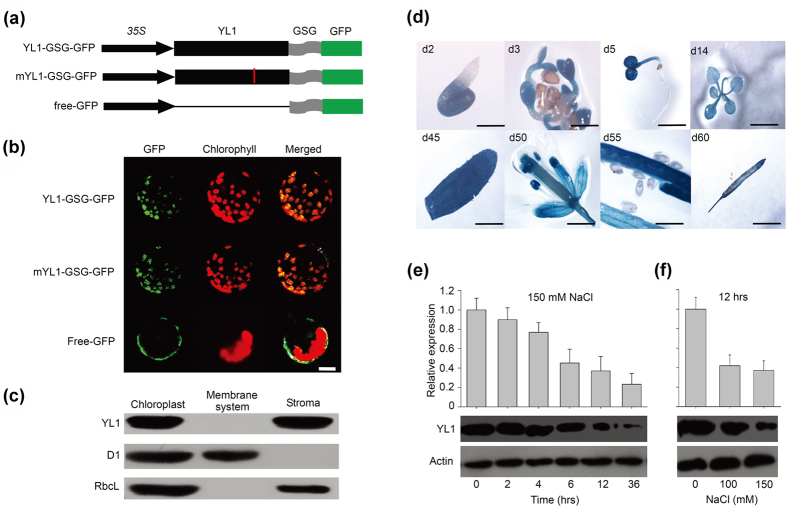
Subcellular Localization and Expression Patterns of YL1. (**a**) The structures of plasmids used for YL1 protein subcellular localization experiment. The black arrows indicate the *35S* promoter; the black cuboids indicate the CDS of *YL1*; the gray curving cuboids indicate the flexible peptide (GSG) and the green cuboids indicate the *GFP* CDS. For plasmid of mYL1-GSG-GFP, the red break line indicates the mutant site (G to A) of *YL1* in *yl1-1* mutant. (**b**) Localization of YL1 protein within the chloroplast by GFP assay. The green fluorescence indicates the GFP, the red indicates the chlorophyll fluorescence. Bar = 5 μm. (**c**) YL1 localizes in the stroma fractions. Intact chloroplasts were isolated from cotyledons of 5-d-old *yl1-1* seedlings, then separated into stroma and membrane fractions, which were used for immunonlot analysis. D1 is mainly associated with membrane system while RbcL localizes in stroma. (**d**) GUS staining analysis of *YL1* in a 2-d-old germinating peeled seed (scale bar = 1 mm), expanded cotyledons of 3-d-old seedlings (scale bar = 5 mm), 5-d-old seedling cotyledons (scale bar = 5 mm), 14-d-old plant leaves (scale bar = 10 mm), a green cauline leaf from a 45-d-old plant (scale bar = 5 mm), a flower with full organs (scale bar = 5 mm), immature green seeds from a 55-d-old plant (scale bar = 1 mm), and a silique containing mature seeds from a 60-d-old plant (scale bar = 1 mm). (**e**) qRT-PCR and immunoblot analysis of YL1 expression levels under salt stress. Total RNA or protein was isolated from 5-d-old seedling cotyledons treated with 150 mM NaCl for different time. (**f**) qRT-PCR and immunoblot analysis of YL1 expression levels under different NaCl concentrations. Total RNA or protein was isolated from cotyledons of 5-d-old seedlings treated with 0 mM, 100 mM, or 150 mM NaCl for 12 h. For immunoblot analysis, each lane was loaded on 20 μg cotyledon protein. For transcriptional analysis, the internal control gene was *Actin2*. Three independent experiments were performed with similar results. One representative experiment is showed.

Previous studies stated that YL1 mainly localizes in chloroplast^31^,^32^. To further determine details of YL1 subcellular localization, we constructed a *YL1-GSG-GFP* plasmid containing a flexible peptide (GGGSSSSGGG) between the *YL1* and *GFP* gene sequences (Fig. 3a). GFP fluorescence was clearly detected in the chloroplasts of *YL1-GSG-GFP* transgenic plant protoplasts (Fig. 3b). Immunoblot analysis of the stroma and membrane fractions from Percoll-purified chloroplasts demonstrated that YL1 protein is localized in the stroma, and not in the membrane (Fig. 3c). Here, the chloroplast stroma protein RbcL and thylakoid membrane protein D1 were used as control (Fig. 3c). In addition, the localization of the mutated YL1 (mYL1) in *yl1-1* is not changed (Fig. 3b), further confirming that the mutation elicits protein function defects.

The expression pattern analysis used in β-glucuronidase (GUS) staining showed that the *YL1* gene is expressed in most of the green tissues throughout the plant growth cycle, even in germinating seeds (Fig. 3d). However, the GUS activity is extremely low in roots and mature seeds (Fig. 3d), suggesting low expression of *YL1* in these tissues. To detect the effect of salt stress on the expression of *YL1*, total RNA was isolated from 5-d-old wild type seedling shoots harvested after salt treatment. The results of qRT-PCR showed that the levels of *YL1* transcripts were evidently reduced with increasing salt concentration or treatment time, which were further confirmed by immunoblot and GUS activity analysis (Figs 3e,f and S3). When plants were treated with 150 mM NaCl for 12 h, *YL1* expression decreased to less than half of that of the non-treated plants (Fig. 3e,f). In addition, no matter treated with or without NaCl, *YL1* transcripts were hardly detected in roots (Fig. S4). These results indicate that the *YL1* could be significantly downregulated by salt stress in seedling shoots.

### Loss of YL1 Function Causes Shoot Na^+^ Accumulation under Salt Stress

**Figure 4:**
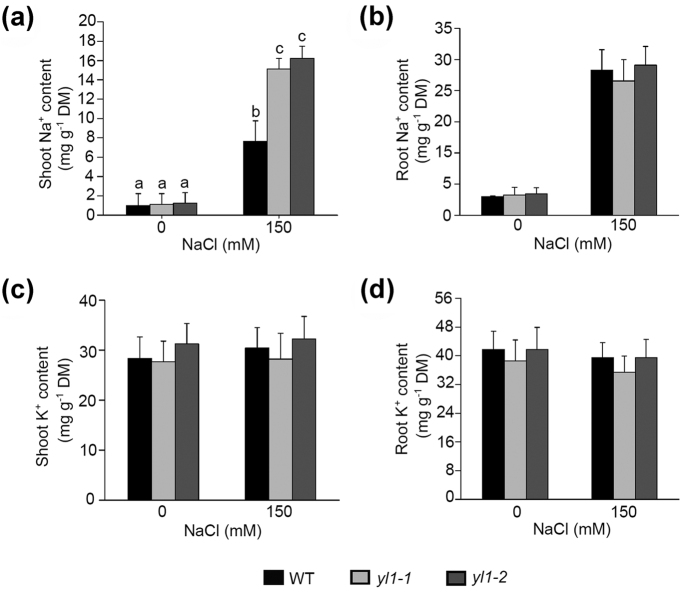
Na^+^ and K^+^ contents in seedling shoots or roots. (**a**) Na^+^ contents in 5-d-old seedling shoots of wild type, *yl1-1*, and *yl1-2* treated with 0 mM or 150 mM NaCl for 2 d on 1/2 MS medium. Data represent means ± SE of three independent experiments. One-way ANOVA (Duncan’s multiple range test) was performed, and statistical significant differences are indicated by different lowercase letters (P < 0.01). (**b**) Na^+^ contents in roots of 5-d-old seedlings described in (**a)**. (**c**) K^+^ contents in shoots of 5-d-old seedlings described in (**a)**. (**d**) K^+^ contents in roots of 5-d-old seedlings described in (**a)**.

Basing on the results presented in Fig. 1b, we speculate that Na^+^ homeostasis is disrupted in *yl1-1* shoot tissues under salt stress conditions. Thus, Na^+^ and K^+^ levels were measured using atomic absorption spectrophotometry. The Na^+^ contents in the shoots were substantially higher in *yl1-1* and *yl1-2* than in wild type plants treated with 150 mM NaCl for 2 days (Fig. 4a). By contrast, no differences in Na^+^ contents were observed in roots among these genotypes (Fig. 4b). K^+^ levels were similar among the three genotypes regardless of tissue type (Fig. 4c,d). Thus, we conclude that Na^+^ is over-accumulated in the shoots of *yl1* seedlings under salt stress.

### Loss of YL1 Function Affects *ABI4* Expression in Seedling Shoots

**Figure 5:**
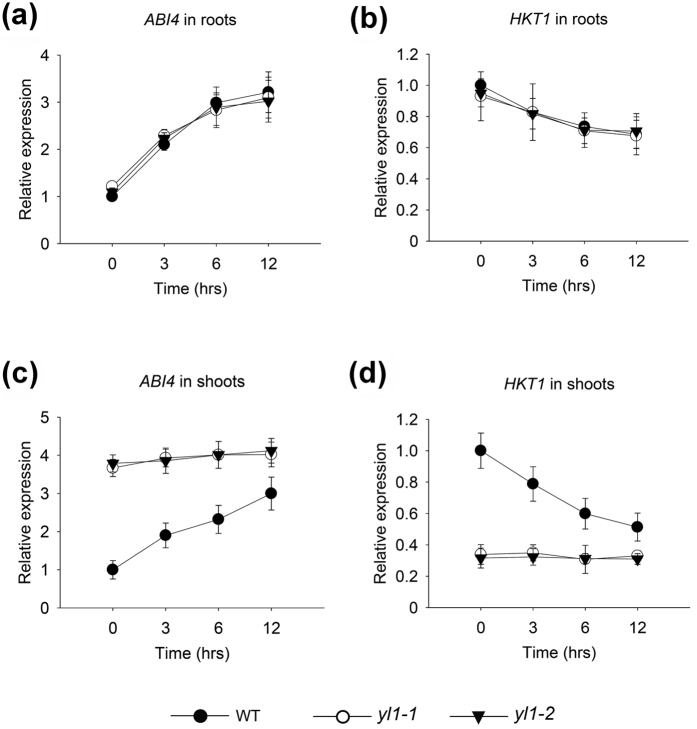
Relative expression analysis of *ABI4* and *HKT1*. (**a**) Transcript abundance analysis of *ABI4* in roots of wild type, *yl1-1*, and *yl1-2* 5-d-old seedlings treated with 150 mM NaCl for 0, 3, 6, or 12 h. (**b**) Transcript abundance analysis of *HKT1* in 5-d-old seedling roots of wild type, *yl1-1*, and *yl1-2* treated with 150 mM NaCl for 0, 3, 6, or 12 h. (**c**) Transcript abundance analysis of *ABI4* in shoots of wild type, *yl1-1*, and *yl1-2* 5-d-old seedlings treated with 150 mM NaCl for 0, 3, 6, or 12 h. (**d**) Transcript abundance analysis of *HKT1* in 5-d-old seedling shoots of wild type, *yl1-1*, and *yl1-2* treated with 150 mM NaCl for 0, 3, 6, or 12 h. The internal control gene was *Actin2*. Data represent means ± SE of three independent experiments.

To identify the factor(s) that possibly play roles in Na^+^ over-accumulation in the shoots of *yl1-1* and *yl1-2*, transcripts of several genes involved in plant salt stress response were detected. The results showed that, regardless of treatment with or without salt, no clear differences were observed in the expression levels of the *SOS* pathway genes, ABA biosynthesis pathway genes, or *NHX1* among the studied genotypes (Figs S5 and S6). Similar results of *ABI4* or *HKT1* transcription levels were found in the roots of the detected genotypes (Fig. 5a,b). However, in the shoots of the mutant seedlings, *ABI4* expression was three- to fourfold higher, whereas *HKT1* transcription levels were substantially lower than those in the wild type grown under normal growth conditions (Fig. 5c,d). Moreover, after 12 h of salt stress treatment, evident inductions of *ABI4* and reductions in *HKT1* transcripts were observed in the shoots of wild type and roots of all detected genotypes (Fig. 5), but no significant changes of ABI4 or *HKT1* could be observed in the mutant shoots (Fig. 5c). These results suggest that loss of YL1 function impairs *ABI4* and *HKT1* expression in seedling shoots. A previous study stated that ABI4-overexpressed plants are sensitive to salt stress because of *HKT1* depression^23^. Based on these evidences, the salt-sensitive phenotype of the *yl1* mutant shoot is possibly tightly associated with the high levels of *ABI4*.

### ABI4 Functions in Salt Stress Response of *yl1* Mutant Seedlings

**Figure 6:**
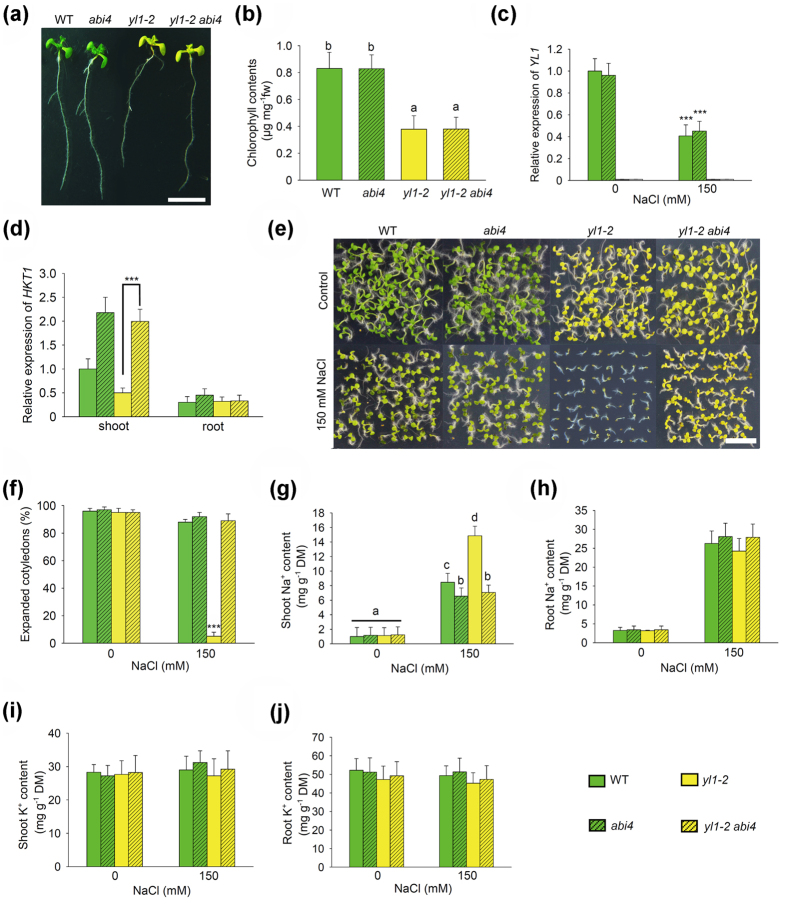
Loss of ABI4 rescues salt stress sensitivity of *yl1* seedlings. (**a**) Phenotypes of 10-d-old wild type, *abi4*, *yl1-2*, and double mutant *yl1-2 abi4* grown under normal growth conditions. Scale bar = 10 mm. (**b**) Chlorophyll contents (chlorophyll a & b) of 10-d-old plants showed in (**a)**. (**c**) Transcript abundance analysis of *YL1* in wild type, *abi4*, *yl1-2*, and double mutant *yl1-2 abi4* 5-d-old seedlings treated with 150 mM NaCl for 12 h or not. (**d**) Transcript abundance of *HKT1* in 5-d-old seedling shoots and roots of genotypes showed in (**a)**. (**e**) 5-d-old seedling phenotypes of wild type, *abi4*, *yl1-2*, and double mutant *yl1-2 abi4* grown in 0 mM or 150 mM NaCl. Scale bar = 5 mm. (**f**) The percentages of the fully expanded cotyledons of plants showed in (**e)**. (**g**) Na^+^ contents in 5-d-old seedling shoots of wild type, *abi4*, *yl1-2*, and double mutant *yl1-2 abi4* treated with 0 mM or 150 mM NaCl for 2 d. (**h**) Na^+^ contents in 5-d-old seedling roots described in (**g)**. (**i**) K^+^ contents in 5-d-old seedling shoots described in (**g)**. (**j**) K^+^ contents in 5-d-old seedling roots described in (**g)**. For (**b,g)**, one-way ANOVA (Duncan’s multiple range test) was performed, and statistical significant differences are indicated by different lowercase letters (P < 0.01). For (**c**,**d**,**f**,) values are means ± SE of three independent replicates (asterisk indicates P < 0.001, Student’s *t*-test). For (**c,d**) the internal control gene was *Actin2*.

To further confirm the above issue, a double mutant, *yl1-2 abi4*, was created. The 10-d-old seedling shoot of *yl1-2 abi4* showed a pale-green phenotype due to the low chlorophyll contents, revealing that the YL1 roles in leaf coloration are independent of ABI4 (Fig. 6a,b). *YL1* transcripts were not clearly influenced by the *abi4* mutant, indicating that *YL1* expression was also ABI4-independent (Fig. 6c). However, *HKT1* mRNA levels were significantly higher in the shoots of *yl1-2 abi4* than in the *yl1-2* single mutant, which was similar to that in *abi4* (Fig. 6d). Furthermore, under high-salinity growth conditions, the percentages of the fully expanded cotyledons of *yl1-2 abi4* seedlings were similar to those of the wild type and *abi4* (Fig. 6e,f). The seedling shoots of salt-treated *yl1-2 abi4* also accumulated lower levels of Na^+^ than the *yl1-2* single mutant, whereas K^+^ contents did not considerably change in all detected genotypes (Fig. 6g–j). Thus far, the abovementioned results clearly indicate that the salt stress-sensitive phenotype of *yl1-2* shoot could be completely rescued by the loss of ABI4 function.

## Discussion

Hyperionic stress causes plant growth inhibition or plant death by disturbing the physiological functions of the shoots and roots^3^,^5^,^25^. This study focused on shoot phenotypes to elucidate the mechanisms of plant response to salt stress. *yl1-1*, a mutant with severely stunted shoot phenotype under salt stress conditions, was isolated from an EMS-mutagenized library in Col-0 background (Fig. 1a). YL1, which is mainly localized in the chloroplast stroma (Fig. 3b,c), is a YqeH-type GTPase. GTPase, including Obg, Era, YlqF, YqhC, YsxC, YqeH, and YloQ-type families, exists in almost all organisms and is involved in regulating diverse cellular processes^33^,^34^. However, the YqeH is only conserved in bacteria and plant chloroplast^35^. In *Bacillus subtilis*, YqeH is involved in 30S ribosome subunit biogenesis and 16S rRNA maturation^36^,^37^. In plants, BPG2 (YL1) plays roles in chloroplast ribosome RNA maturation^31^. Kim *et al*. proposed that BPG2 (YL1) binds to plastid rRNA for chloroplast translation apparatus assembly^32^. AtNOA1/RIF1, a YqeH-type GTPase homolog in *Arabidopsis*, confers an atypical nitric oxide synthase activity, plastid ribosome function, and root growth behavior control^38^,^39^,^40^. OsNOA1 RNAi rice seedlings show chlorotic phenotype^41^. Nevertheless, no study has investigated the involvement of YqeH-type proteins in environmental stress response.

Na^+^ accumulation was high in the shoots of *yl1-1* and *yl1-2* under salt stress conditions (Fig. 4a), suggesting that the mechanisms of Na^+^ exclusion were interrupted because of the loss of YL1 functions. Na^+^ exclusion broadly refers to two mechanisms: Na^+^ efflux from root epidermis and decrease of Na^+^ delivery from root to shoot^4^. SOS pathway genes play vital roles in Na^+^ efflux in roots^9^,^42^. Homologs of SOS1 from rice, wheat, and tomato have been characterized in controlling Na^+^ transport^43^,^44^,^45^. YL1 was difficult to detect in roots, and the expression levels of *SOS* pathway genes were not altered in the *yl1* mutants (Figs 3a and S6). Moreover, loss of YL1 functions did not clearly affect root development or ion homeostasis under controlled or high salinity conditions (Figs 4 and S1). These results suggest that the salt hypersensitivity of mutant shoots is not caused by the reduced Na^+^ efflux from root epidermis but probably by the increase of Na^+^ delivery from root to shoot. To our knowledge, Na^+^ delivery from root to shoot mainly depends on the transpiration stream in the xylem^4^,^46^,^47^. HKT1 could directly retrieve Na^+^ from the xylem sap back to the phloem of the shoot and unload it in the root, which in turn reduces Na^+^ accumulation in the shoot^13^,^15^. In wheat, TmHKT1;4-A2 is expressed in leaf sheath and reduces Na^+^ concentration in the shoot^48^. *ABI4* has been recently reported to act as a negative regulator that could directly bind to the promoter region and inhibit *HKT1* expression in *Arabidopsis*^23^. In the shoots of *yl1* mutants, *ABI4* was highly expressed, and the *HKT1* transcription levels were approximately half of those of the wild type (Fig. 5). By contrast, in the shoots of the *yl1-2 abi4* double mutant, the transcripts of *HKT1* were evidently higher than those in *yl1-2* (Fig. 6d). Furthermore, the loss of ABI4 function could rescue *yl1-2* shoot salt-sensitive phenotypes (Fig. 6e,f). These observations suggest that *HKT1* is depressed by high levels of *ABI4* in shoots of *yl1* mutants.

ABI4, which binds to the coupling element 1 (CE1, CACCG) *in vitro*, is involved in regulating a large number of genes^49^,^50^. ABI4 plays key roles in sugar, ABA, and pathogen-response signaling pathways through binding to S-box or G-box sequences^51^,^52^. Furthermore, *ABI4* expression could be regulated by many factors. During seed maturation, germination, and the early-stage of seedling development, ABI4 could be induced by ABA and cytokinin and be repressed by auxin^53^,^54^. ABI4 could also directly bind to its own promoter CE1-like element and activate gene expression^55^. Previous studies also reported that ABI4 is significantly downregulated in the *wrky18* and *wrky40* mutant seedlings^56^. Moreover, the transcription factor SCR was also proven to directly bind to *ABI4* promoter and negatively regulate *ABI4* expression^57^.

At the seedling stage, similar to *YL1*, *ABI4* expression is readily detected in shoots^55^. Our results showed that *ABI4* was more highly expressed in the shoots of *yl1* mutants compared with in the shoots of the wild type (Fig. 5c), whereas in the roots of *yl1-1* or *yl1-2*, ABI4 expression was almost unchanged (Fig. 5a). Consistent with previous study^23^, *ABI4* transcripts could be clearly increased by salt stress signaling in the wild type (Fig. 5c), which is contrary to *YL1* (Fig. 3e,f). However, in shoots of *yl1*, the salinity effect on *ABI4* expression was clearly weakened (Fig. 5c). The abovementioned results suggest that YL1 may be a potential factor that acts upstream of ABI4 in salt stress signaling because the *YL1* expression is independent of ABI4 (Fig. 6c). Together with the phenotype of *yl1-2 abi4* under salt stress treatment (Fig. 6e), we reveal that ABI4 plays roles in seedling shoot salt-sensitive phenotype of *yl1* mutants. Considering that the YL1 protein localizes in the chloroplast and ABI4 is a nucleus-localized transcription factor, several signals may be derived from the chloroplast and effectively transmitted to the nucleus when YL1 dysfunctions. Several studies stated that ABI4 plays key roles in chloroplast retrograde signaling. ABI4, which could bind the *Lhcb* family gene promoter CCAC-motif, acts downstream of GUN1 and PTM and negatively regulates target gene transcriptions^20^,^22^,^58^. Thus, whether YL1 regulates ABI4 through retrograde signaling need to be identified in future studies.

Recent studies have identified several genes are involved in plant shoot response to salt stress^46^,^47^. Nevertheless, the regulatory mechanisms of plant shoot salt tolerance remain unclear. *yl1-1* was investigated, and the results suggested that chloroplast proteins such as YL1 could be involved in plant salt stress response through nuclear stress-responsive factors. We speculate that the reduction of *YL1* and induction of *ABI4* under high salinity conditions may be an adaptive mechanism to achieve Na^+^ equilibrium in the entire plant, which needs to be investigated further. Although the signaling pathway is unclear, our results may open a new insight into the association of plant salt stress response and the chloroplast.

## Methods

### Plant Materials and Growth Condition

All wild type plants in the study are *Arabidopsis thaliana* Col-0. The mutants, *yl1-1*, *yl1-2* (salk_068713), and *abi4* (salk_080095) are in the Col-0 genetic background. NaClO (0.1%) and ethanol (70%) are used for seed sterilization. Seedlings are grown in solid 1/2 Murashige and Skoog (MS) medium containing 1% sucrose.

### Constructs and plant transformation

For construct used in *yl1-1com*, CDS sequence of *YL1* was subcloned into pBI121 vector with recognition sites for the restriction enzymes BamHI and SalI. For *YL1-GSG-GFP* (*mYL1-GSG-GFP*) construct, a flexible peptide GSG (GGGSSSSGGG) was added between *YL1* (*mYL1*) CDS sequence of termination coden free and GFP sequence. The *YL1-GSG-GFP* (*mYL1-GSG-GFP*) sequence was also cloned into pBI121 vector. Constructs were introduced into *Agrobacterium tumefaciens* GV3101 for plant transformation. Transgenic plants were selected on 1/2 MS medium containing 50 mg L^−1^ kanamycin. Plants were selfed twice and T3 homozygous plants were used. Primers are listed in Table S1.

### Stress Treatment and Phenotypes Recording

Surface sterilized seeds were plated in solid 1/2 MS medium described above containing 150 mM NaCl or not. Plated seeds were 4 °C-dark treated for 2 d, then transferred to incubator (22 °C, 16/8 h light/dark) and grown for 5 d. The number of plants with (n) or without (m) fully expanded cotyledons was recorded. Then the percentages of fully expanded seedling cotyledons were calculated with (n/n + m) *100%.

### Transcript Analysis

For gene transcription analysis under salt conditions, 4-d-old seedlings grown under normal conditions were transferred to the MS medium containing 0, 100, or 150 mM NaCl and then treated for different times (2–36 h). The shoots and roots were separately harvested and total RNA was extracted with Trizol (invitrogen). cDNA synthesis was performed using PrimeScript reverse transcriptase (RT) with oligo dT primer using the PrimeScript RT master mix kit (Takara). qRT-PCR experiments were carried out by an CFX96 real-time PCR system (Bio-Rad, C1000) using SYBR green real-time PCR master mix (Takara). Detection for each gene transcript was performed for at least three biology replicates and each bio-replicate containing three technical replicates. All used primers are listed in Table S1.

### *YL1* Promoter Construction and GUS staining

The *pYL1-GUS* was constructed by subcloning a 1.5 kb fragment upstream of the *YL1* translation start site into pBI122 binary vector. GUS staining analysis for different tissues and GUS activity analysis in seedlings for different treatment were according to Yan^59^. Fluorescence of GUS was measured with a Microplate Spectrofluorometer (IC Measurement Acc for FL Solutions, F-4500, HITACHI). Primers are listed in Table S1.

### Immunoblot Analysis

Plant materials were prepared according to the methods described in Transcript analysis. Total proteins of such as seedling shoots were extracted using Plant Protein Extraction Kit (CWBIO) and measured using a nano-drop instrument (Nano-Drop, ND-1000 Spectrofluorometer). The polyclonal antibody of YL1 was obtained from rabbits by Abmart (China). The RbcL, D1 and Actin antibodies were all purchased from Agrisera. Proteins after electrophoresis were blotted to nitrocellulose membranes and then probed with specific antibodies. The nitrocellulose membranes were visualized with an enhanced Lumi-Light Western Blotting Substrate kit (Thermo Scientific).

### Ion Content Measurement

For measurement to Na^+^ and K^+^. the 3-d-old seedlings grown under normal conditions were transferred to the MS medium containing 0 or 150 mM NaCl and then grown for 2 d. The shoots and roots from these 5-d-old plant were separately harvested, dried for 48 h at 80 °C and then ground to powder. The same mass tissue powder was digested in concentrated (69%, v/v) HNO_3_ for 24 h at room temperature for elemental extraction. Na^+^ and K^+^ concentrations was determined by atomic absorption spectrophotometry (novAA300, analytikjena).

### Chloroplast Isolation

5-d-old seedling cotyledons were harvested and grounded in isolation buffer (20 mM HEPES/KOH, pH 8.0, 0.3 M sorbitol, 5 mM MgCl_2_, 5 mM EGTA, 5 mM EDTA, and 10 mM NaHCO_3_). The homogenate was filtered and centrifuged at 3000 *g* for 3 min. The pellet was resuspended in 1 mL isolation buffer. The intact chloroplasts are isolated through Percoll gradient method^60^. Thylakoid membranes and stroma proteins were prepared from isolated intact chloroplasts.

### Chlorophyll content measurement

Cotyledons or leaves of plant were collected, fresh weighed and washed in distilled water. Chlorophyll was extracted in 80% (v/v) acetone at 25 °C in darkness for 24 h, and concentration of chlorophyll a/b was determined according to Komatsu^31^.

### Accession Numbers

Sequence data from this article can be found in the *Arabidopsis* Genome Initiative or GenBank/EMBL data libraries under the following accession numbers: *YL1*, At3g57180; *ABI4*, At2g40220; *HKT1*, At4g10310; *SOS1*, At2g01980; *SOS2*, At5g35410; *SOS3*, At5g24270; *NHX1*, At5g27150; *ABA1*, At5g67030; *ABA2*, At1g52340; *ABA3*, At1g16540; *AAO3*, At2g27150; *NCED3*, At3g14440.

## Additional Information

**How to cite this article**: Li, P.-C. *et al. Arabidopsis* YL1/BPG2 Is Involved in Seedling Shoot Response to Salt Stress through ABI4. *Sci. Rep.* **6**, 30163; doi: 10.1038/srep30163 (2016).

## Supplementary Material

Supplementary Information
