# Supplementary material for: Arabidopsis YL1/BPG2 Is Involved in Seedling Shoot Response to Salt Stress through ABI4
Source: Sci Rep. 2016 Jul 22;6:30163. doi: 10.1038/srep30163 (PMC4957093; doi:10.1038/srep30163)
Supplement: Supplementary Information [file srep30163-s1.pdf]

Title: *Arabidopsis* YL1/BPG2 Is Involved in Seedling Shoot Response to Salt Stress through ABI4

Authors: Peng-Cheng Li[1,2†], Jin-Guang Huang[1†], Shao-Wei Yu[1], Yuan-Yuan Li[1], Peng Sun[1], Chang-Ai Wu[1], and Cheng-Chao Zheng[1*]

Affiliations:

[1]State Key Laboratory of Crop Biology, College of Life Sciences, Shandong Agricultural University, Tai'an, Shandong 271018, PR China

[2]High-Tech Research Center, Shandong Academy of Agricultural Sciences, Shandong Provincial Key Laboratory of Crop Genetic Improvement, Ecology and Physiology, Jinan, Shandong, PR China

[†]These authors contributed equally to this work.

*Correspondence should be addressed to C-C.Z. (e-mail: cczheng@sdau.edu.cn)

Supplementary information

Supplementary Figure 1. Root phenotype of *yl1-1*.

Supplementary Figure 2. Mature plant phenotypes of wild type, *yl1-1*, *yl1-2*, *yl1-1com*, and *yl1-1 yl1-2* (cross F1 line).

Supplementary Figure 3. GUS activity analysis of the *pYL1-GUS* transgenic plants under salinity treatment.

Supplementary Figure 4. Transcriptional analysis of *YL1* in roots under salt-treatment.

Supplementary Figure 5. Transcriptional analysis of SOS pathway genes, *NHX1*, and ABA biosynthesis pathway genes in seedling shoots.

Supplementary Figure 6. Transcriptional analysis of SOS pathway genes, *NHX1*, and ABA biosynthesis pathway genes in seedling roots.

Supplementary Table 1. Key primers used in this study.

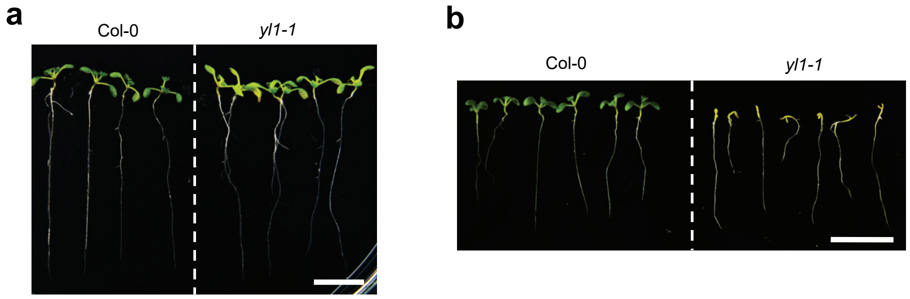

Supplementary Figure 1. Root phenotype of *yl1-1*.

(**a**) Phenotype of 10-d-old seedling roots under control conditions. Scale bars = 5 mm.

(**b**) Phenotype of 10-d-old seedling roots under 150 mM NaCl conditions. Scale bars = 5 mm.

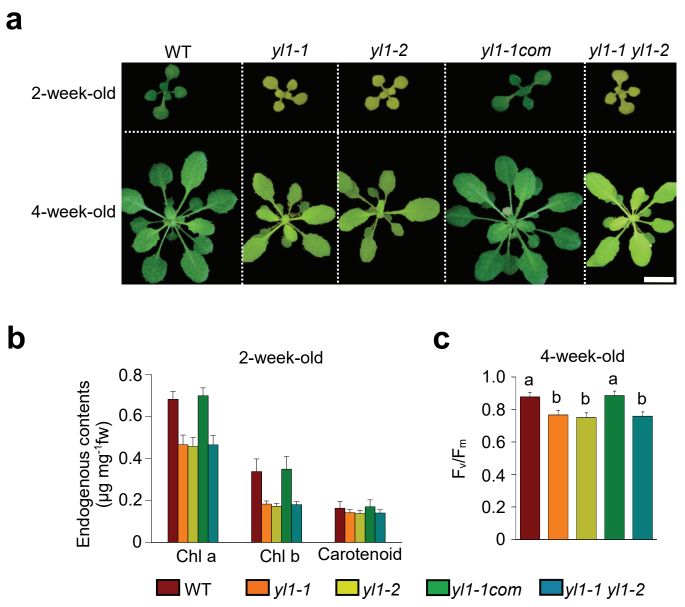

Supplementary Figure 2. Mature plant phenotypes of wild type, *yl1-1*, *yl1-2*, *yl1-1com*, and *yl1-1 yl1-2* (cross F1 line).

(**a**) Phenotypes of 2/4-week-old wild type, *yl1-1*, *yl1-2*, *yl1-1com*, and *yl1-1 yl1-2* (F1) grown under normal growth conditions. Scale bars = 10 mm.

(**b**) Endogenous contents of chlorophyll a, chlorophyll b, and carotenoid in 2-week-old wild type, *yl1-1*, *yl1-2*, *yl1-1com*, and *yl1-1 yl1-2* (F1).

(**c**) F_v/F_m of 4-week-old wild type, *yl1-1*, *yl1-2*, *yl1-1com*, and *yl1-1 yl1-2* (F1).

Data are mean values of three replicates ± SE. Statistical significant differences are indicated by different lowercase letters ($P < 0.01$).

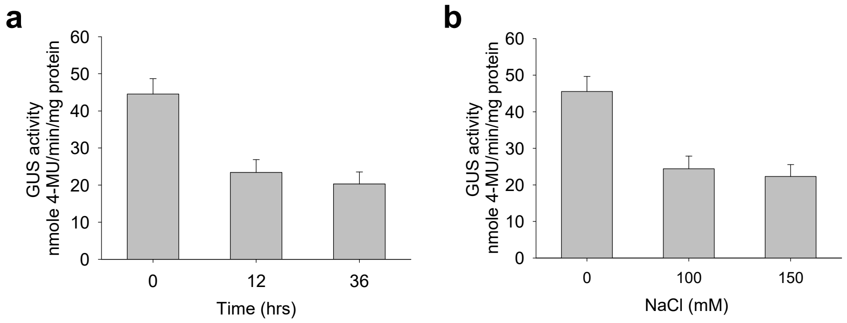

Supplementary Figure 3. GUS activity analysis of the *pYL1-GUS* transgenic plants under salinity treatment.

(**a**) GUS activity analysis of 5-d-old seedlings treated with 150 mM NaCl for 0, 12, or 36 h.

(**b**) GUS activity analysis of 5-d-old seedlings treated with 0, 100, or 150 mM NaCl for 36 h.

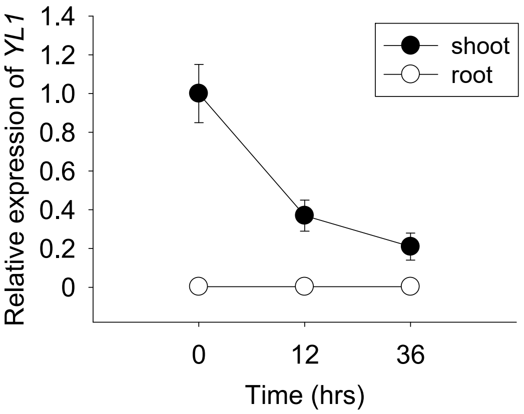

Supplementary Figure 4. Transcriptional analysis of *YL1* in roots under salt-treatment.

Total RNA was isolated from roots of 5-d-seedlings treated with control or 150 mM NaCl for 0, 12, or 36 h. Values are means ± SE of three independent replicates.

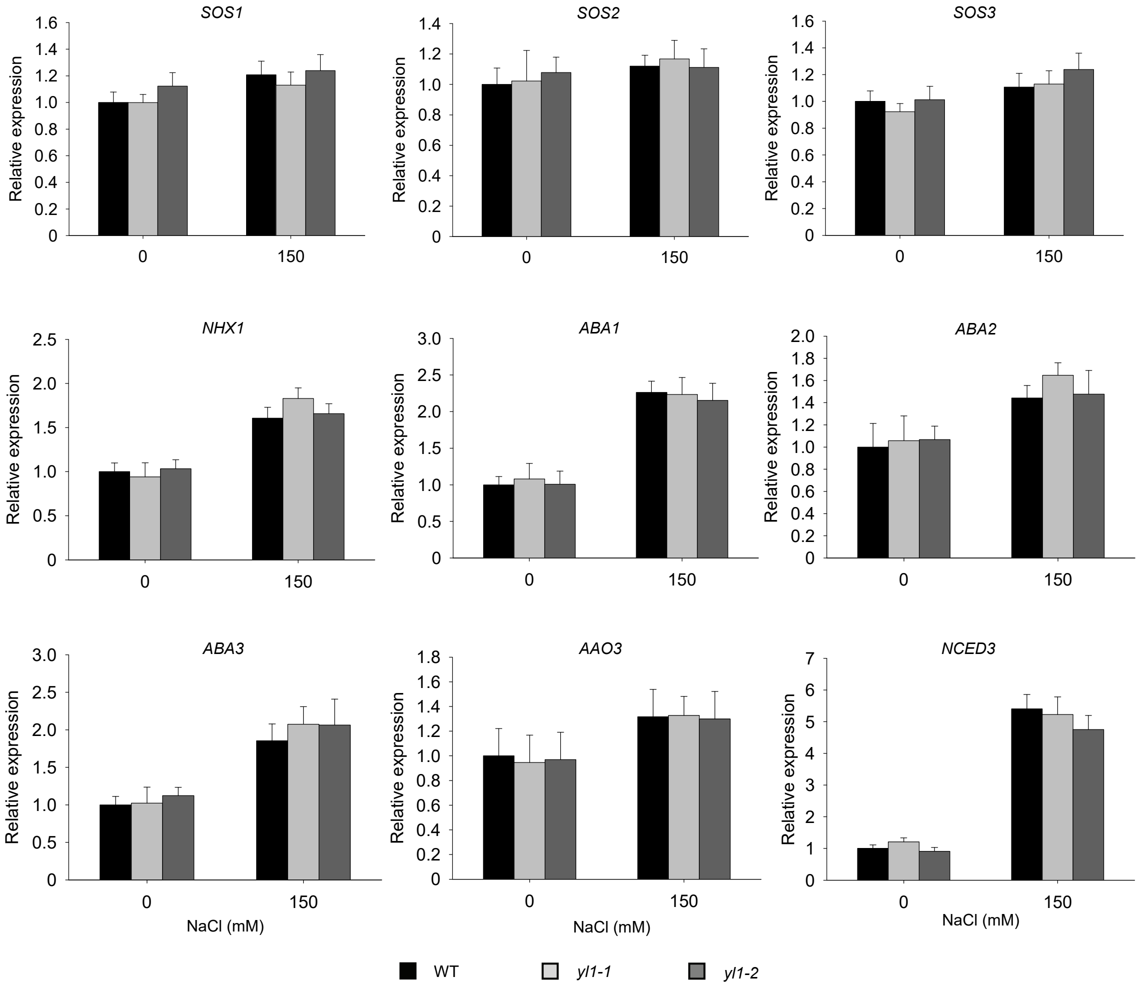

Supplementary Figure 5. Transcriptional analysis of SOS pathway genes, *NHX1*, and ABA biosynthesis pathway genes in seedling shoots.

SOS1, *SOS2*, and *SOS3* are SOS pathway genes; *ABA1*, *ABA2*, *ABA3*, *AAO3*, and *NCED3* are ABA biosynthesis associated genes. Total RNA was isolated from shoots of 5-d-seedlings treated with control or 150 mM NaCl for 12 h. Values are means ± SE of three independent replicates.

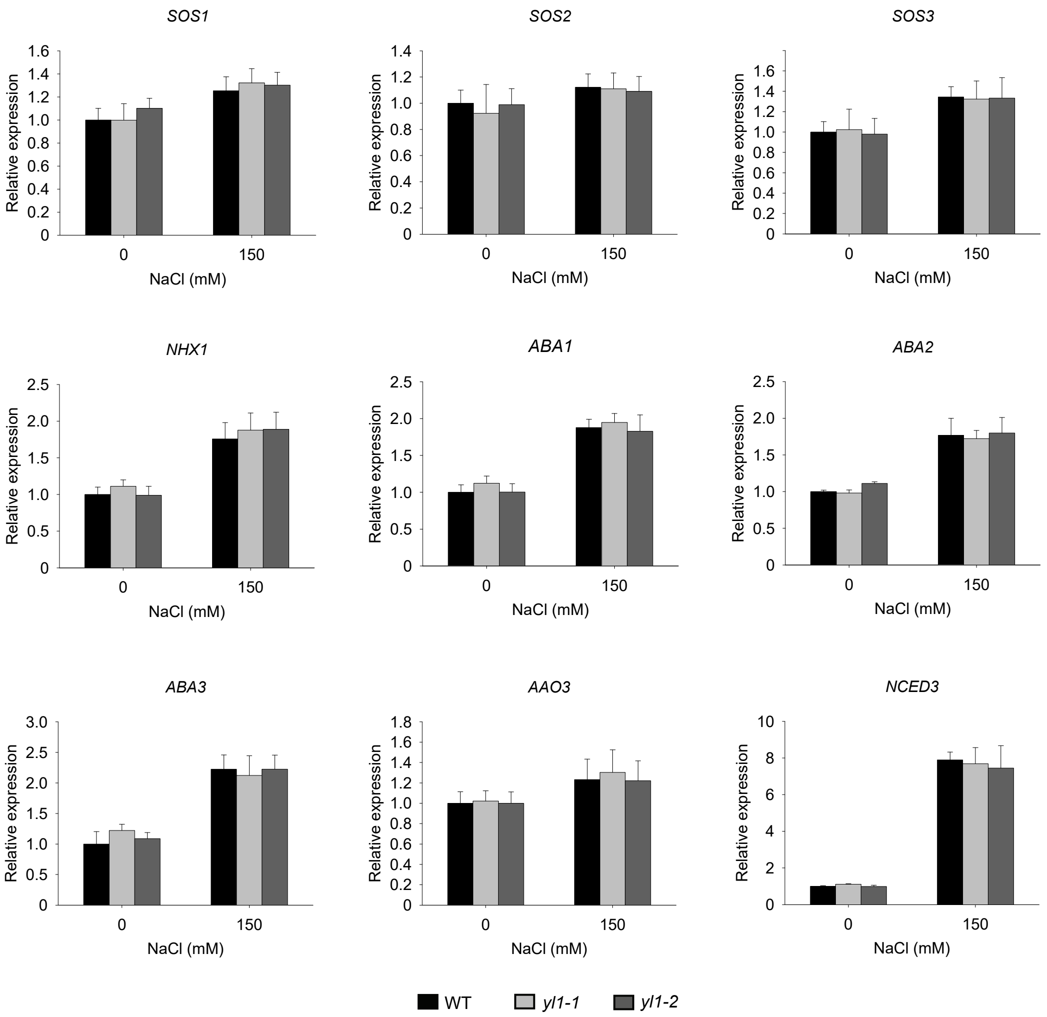

Supplementary Figure 6. Transcriptional analysis of SOS pathway genes, *NHX1*, and ABA biosynthesis pathway genes in seedling roots.

SOS1, *SOS2*, and *SOS3* are SOS pathway genes; *ABA1*, *ABA2*, *ABA3*, *AAO3*, and *NCED3* are ABA biosynthesis associated genes. Total RNA was isolated from roots of 5-d-seedlings treated with control or 150 mM NaCl for 12 h. Values are means ± SE of three independent replicates.

Supplementary Table 1. Primers used in this study.

Purpose	Primer name	Sequence (5'-3')
Complementation and overexpression constructs	*YL1*-forward (P1)	GGATCCATGGTGGTTTTGATTTCAAGTACAG
	YL1-reverse (P2)	TGCGACCTAAGCAACACTATCAGAGAGAAAATC
GFP expression constructs	*YL1*-forward	TCTAGAATGGTGGTTTTGATTTCAAGTACAGTG
	YL1-reverse	GGATCCAGCAACACTATCAGAGAGAAAATCTGT
	GFP-forward	GGATCCGGTGGTGGTAGTAGTGGTGGTGG-<br>-TATGAAGACTAATCTTTTTCTCTTTCTC
	GFP-reverse	GTCGACTTAAAGCTCATCATGTTTGTATAGTTC
GUS expression constructs	*YL1*-promoter-forward	GTCGACATGGTGGTTGATTTCAAGTACAG
	YL1-promoter-reverse	GGATCCAGCAACACTATCAGAGAGAAAATC
qRT- PCR	*ABA1*-forward	TGCTACTCTATCAATCCATCTCC
	ABA1-reverse	TCTTCTCCTCCTTCTCAACTAAC
	ABA2-forward	AGGAGCCACAGGGATAGG
	ABA2-reverse	ACTCACCACGAAGCAGAC
	ABA3-forward	CCGAGGAGAGTGTTGCTGAC
	ABA3-reverse	AACCGCCCAATGATGTGAAATG
	AAO3-forward	TGCTTATGGTCTCGGTATGG
	AAO3-reverse	CCTAACGGCTTCACAACTG
	NCED3-forward	GGTCGTGTGAGTTCTTATGTG
	NCED3-reverse	ACTGGTAAATCTCGCTCTCTC
	ABI5-forward	AACCTAATCCAACCCGAACC
	ABI5-reverse	ACCCTCCTCCTCCTGTCC
	ABI4-forward	CTCTTCCTCCTCCGTCTC
	ABI4-reverse	GTTGGCTCCTCCTCCTAC
	HKT1-forward	TCTGGTCTCATCTGGCTCCTAATC
	HKT1-reverse	CACCGTCACTCCAAGAAGAACAC
	SOS1-forward	TGGAGGAAGCGACCGATTC
	SOS1-reverse	CGATAACGAGAAGAGCGACAG
	SOS2-forward	CTGTTGCTCATTGTCACTG
	SOS2-reverse	CGTAACCCTGTCCACTAAG
	SOS3-forward	GTCACGCCATTCACGGTAG
	SOS3-reverse	CACTCCATTTCGCTTCACATC
	NHX1-forward	AGGTGCTGTATCTATGGCTCTTG
	NHX1-reverse	GCTCGTGGTGGCGTTCTG
	YL1-forward (qP1)	GGATTGATGTAACCTTGAGAG
	YL1-reverse (qP2)	GCTGTCTTGTTGCTTCTTC
	Actin2-forward	CTTCGTCTTCCACTTCAG
	Actin2-reverse	ATCATACCAGTCTCAACAC
